# Immobilizing Laccase on Modified Cellulose/CF Beads to Degrade Chlorinated Biphenyl in Wastewater

**DOI:** 10.3390/polym10070798

**Published:** 2018-07-19

**Authors:** Na Li, Quiyang Xia, Yuan Li, Xiaobang Hou, Meihong Niu, Qingwei Ping, Huining Xiao

**Affiliations:** 1 Liaoning Province Key Laboratory of Plup and Papermaking Engineering, Dalian Polytechnic University, Dalian 116034, China; lina@dlpu.edu.cn (N.L.); niumh@dlpu.edu.cn (M.N.); pingqw@dlpu.edu.cn (Q.P.); 2 Key Laboratory of Pulp and Paper Science & Technology of Ministry of Education/Shandong Province, Qilu University of Technology, Jinan 250353, China; 3 Department of Chemical Engineering, University of New Brunswick, Fredericton, NB E3B 5A3, Canada; qxia2@unb.ca (Q.X.); yli7@unb.ca (Y.L.); 4 Department of Environment Science & Engineering, North China Electric Power University, Baoding 071003, China; xhou1@unb.ca

**Keywords:** chlorinated biphenyl pollutants, cellulose/CF beads, adsorption, laccase immobilization

## Abstract

Novel modified cellulose/cellulose fibril (CF) beads (MCCBs) loaded with laccase were prepared to degrade polychlorinated biphenyls (PCBs) in wastewater. The proper porous structure in MCCBs was achieved by introducing nano CaCO_3_ (as a pore forming agent) in cellulose/CF (CCBs) beads during the preparation process. Cellulose/CF composite beads were modified by maleic anhydride to introduce carboxyl groups. Laccase was immobilized on the MCCBs through electrostatic adsorption and covalent bonding. The effects of pH, laccase concentration and contact time on immobilization yields and recovered activity were investigated. The best conditions were pH 4, concentration 16 g/L and contact time 3 h. The immobilized laccase under these conditions showed a good performance in thermal and operational stability. The laccase immobilized on MCCB beads can remove 85% of 20 mg/L 4-hydroxy-3,5-dichlorobiphenyl (HO-DiCB) in wastewater. The results demonstrated that MCCBs, as a new type of green-based support, are very promising in material immobilizing laccase. This technology may be of potential advantage for the removal of polychlorinated biphenyls in wastewater from an environmental point of view.

## 1. Introduction

Polychlorinated biphenyls (PCBs) are a class of materials commonly used in various commercial products. These materials can cause environmental contaminations both due to its toxicity and stability against degradation in soil and water [1], and are included in the Persistent Organic Pollutions (POP) group [2]. In the past decade, great attention has been focused on the degradation of PCBs due to their toxicity and bioaccumulation [3]. Several methods have been developed to degrade PCBs, including the degradation induced by laccase, which was considered as an efficient route to solve the problem because the phenolic compounds were the major substrates of laccase [4,5]. Although laccase showed a good performance in the removal of PCBs [6], some negative characteristics, such as instability caused by thermal and pH denaturation, poor reusability and inactivation, have hindered the application of laccase in water treatment [7,8,9]. In order to overcome such shortcomings, enzyme immobilization, one of the most promising techniques for highly efficient and economically competent biotechnological processes [10], has been developed.

Laccase immobilization has been researched for a number of years and several materials have been studied as supports [11]. The materials used as supports are often classified into two categories: inorganic materials and organic materials. Among the most abundant organic materials, cellulose is considered promising due to its renewability, easy biodegradation and low contaminant risk to the environment [12,13]. In addition, various reactive groups can be easily introduced onto the cellulose surface [14,15]. Such properties make cellulose suitable for the immobilization of laccase [16]. Immobilization of laccase on bacterial cellulose [17,18] and cellulose nanofibers has been reported [19]. However, in order to facilitate the application of immobilized laccase, other factors should also be considered, such as shape, distribution, pore size and expandability of laccase [20].

In this work, a cellulose-based porous bead MCCBs was used as a novel support for immobilizing laccase. As green and degradable materials, the cellulose and cellulose filament (CF) are chosen to produce the composite-type bioadsorbent for improved mechanical properties and durability. During the beads formation process, CF was added as a reinforcement, and proper pore structure was generated using nano CaCO_3_ as a pore forming agent; additional –COOH groups were introduced via grafting maleic anhydride. The beads with porous structure and functional groups can not only adsorb but also be covalently bonded with laccase. The effects of the immobilization conditions on the immobilization yield and recovered activity were investigated. In addition, 4-hydroxy-3,5-dichlorobiphenyl (HO-DiCB) was used as a model of PCBs to test the ability of immobilized laccase for the removal of PCBs in wastewater.

## 2. Materials and Methods

### 2.1. Materials

Disodium hydrogen phosphate dodecahydrate (Na_2_HPO_4_·12H_2_O), anhydrous citric acid, sodium hydroxide and 2,2’-azino-bis(3-ethylbenzothiazoline-6-sulfonic acid) (ABTS), coomassie brilliant blue (CBB), 95% ethyl alcohol, 85% phosphoric acid (H_3_PO_4_), sodium chloride (NaCl), and bovine serum albumin (BSA) were all purchased from Sigma chemical (Oakville, ON, Canada) and were analytical grade. HO-DiCB was purchased from AccuStandard, New Haven, CT, USA. Laccase (800 U/g) was kindly donated by a Taiwan biological company in Taipei, Taiwan. CF was kindly donated by FPInnovations Canada (Pointe Claire, Montreal, QC, Canada), which was in micro-range (lengths 50–800 μm, surface area >80 m^2^/g). Filter paper is Fisher brand^®^ (Qualitative P4, porosity: Medium–Fine; Flow Rate:Slow, Fisher Scientific, Ottawa, ON, Canada).

### 2.2. Preparation and Modification of the Cellulose/CF Beads

Cellulose beads were prepared using a previously developed method [21,22]. Briefly, 2 g of filter paper was dissolved in NaOH (7 wt %)/Urea (12 wt %) aqueous solution at −10 °C, forming a 4% cellulose solution. 0.8 g nano CaCO_3_ powder and 2 g CF (as a reinforcement) were added into the cellulose solution and the mixture was injected into 1 M HCl solution through a syringe. The CaCO_3_ reacted with HCl, releasing CO_2_ that created a porous structure within dissolved cellulose matrix_._ Finally, the porous Cellulose/CF composite beads [23] were separated from the suspension by filtration and dried at 50 °C in a vacuum oven overnight. 

To modify Cellulose/CF beads, 2 g of Cellulose/CF beads were added into 20 mL of 10 wt/v% maleic anhydride solution in acetone. The reaction was kept for 1 h at room temperature. Then, the mixture was put in 50 °C vacuum oven for 1 h to evaporate the acetone. After evaporation, the residual solids were heated in 100 °C vacuum oven for 3 h and modified Cellulose/CF (MCCBs) were obtained.

### 2.3. Characterization of MCCBs

The chemical bonds of MCCBs were analyzed with Fourier Transform Infrared Spectroscopy (FTIR), and the amount of the carboxyl groups (C=O) on the MCCBs was evaluated using an acid-base titration method. A scanning electron microscopy (SEM) was used to visualize the surface morphology of MCCBs (JEOL 6400, JEOL, Tokyo, Japan). The MCCBs were first inbeded in epoxy resin and then split in half in order to achieve the cross-section. The sliced samples were coated with gold prior to the measurement.

### 2.4. Determination of Laccase Activity

A 0.4 mM ABTS solution was used as substrate to measure the enzyme activity of free and immobilized laccase and all operations were proceeded at 25 ± 1 °C. Free enzyme activity was determined by adding 0.1 mL laccase solution to 1.9 mL ABTS solution and measuring the absorbance of the mixture solution at 420 nm (ε_420_ = 36,000 M^−1^ cm^−1^) [24]. A UV-Vis spectrophotometer (Genesys 10-S, Thermo Electron Corporation, Waltham, MA, USA) was used to measure the increase of the absorbance for 5 min and the absorbance data were recorded per 30 s. Recorded data were plotted into kinetic curve and the activity was determined by calculating the slope of initial linear portion of the curve. Unit of free enzyme activity was expressed in U/L and one U was defined as the amount of free enzyme required to catalyze 1 μmol of substrate per minute.

To measure immobilized enzyme activity, 0.1 g of laccase-loaded support was put in 2 mL of ABTS and 7.0 mL of citrate-phosphate buffer mixed solution at 25 ± 1 °C. For every two minutes, 2 mL of the mixed solution was withdrawn to analyze absorbance by the UV-Vis spectrophotometer at 420 nm and was rapidly returned back to reactor after measurement. U/g was used to express the immobilized enzyme activity per unit gram of support, given that enzyme weight is negligible.

### 2.5. Immobilization of Laccase

The conditions for immobilizing laccase on MCCBs were optimized first. A total of 10 mg of MCCBs were incubated in 10 mL of laccase solution containing 0.5–32 g/L of enzyme in a 20 mL bottle at varying pH from 3.0 to 7.0 in the room temperature. The bottles with the solutions were stirred on a magnetic stirrer. At the optimized conditions of pH and enzyme concentration, the effect of contact time was evaluated in the range from 30 min to 8 h. After immobilization, the support was collected by filtration and washed three times with 0.1 M of phosphate buffer (pH 4.0) (±100 mL each wash). The filtrate was kept for enzyme activity measurements. The immobilized enzyme activity was measured as described above. The immobilization yield was calculated as follows:

(1) $$immobilization=\frac{Originalenzymeconcentration-Enzymeconcentrationafterimmobilization}{Originalenzymeconcentration}\times 100$$

Measurement of enzyme concentration was according to the Bradford method [25].

The recovered activity was calculated using the following equation:

(2) $$recoveredactivity=\frac{immobilizedenzymeactivity}{activityofsameamountoffreeenzyme}\times 100$$

An electron dispersive spectroscopy (EDS) (JEOL 2011, JEOL, Tokyo, Japan) was used to perform an elemental analysis of the immobilized laccase MCCBs.

### 2.6. Thermal and Operational Stability of Immobilized Laccase

The enzyme activities of the free and immobilized laccase were measured to evaluate the thermal stability in pH 4.0 buffer solution at 60 °C. A water bath with temperature controller was used in this experiment. The enzyme activities were measured each hour and the experiment lasted for 6 h. The initial activity was set as 100%.

A total of 0.1 g of laccase-loaded MCCBs was used to measure the operational stability in 2 mL ABTS and 7 mL citrate-phosphate buffer at pH 4.0 and 25 ± 1 °C. The stability experiments were operated for 10 cycles. The immobilized laccase enzyme activity was measured at each cycle and the supports were separated by filtration and washed twice by 30 mL buffer before next cycle.

### 2.7. Degradation of the PCBs

In order to prepare a HO-DiCB aqueous solution, 10 mg of HO-DiCB was reacted with 1.7 mg of NaOH in 50 mL of deionized water; the final volume was adjusted to 250 mL. The degradation efficiency was determined by adding 50 mg immobilized laccase in 10 mL of HO-DiCB aqueous solution and 10 mL of pH 4.0 phosphate buffer at room temperature. The reaction lasted for 8 h and the concentration of HO-DiCB was measured each hour. A control experiment, in which 50 mg CCBs without immobilized laccase was reacted with another 10 mL of PCBs model solution under the same conditions, was carried out simultaneously. The concentration of HO-DiCB aqueous solution was determined by standard curve of absorption at 260 nm. Degradation yield (%) was defined as follows:

(3) $$degradation=\frac{originalHO-DiCBamount-finalHO-DiCBamount}{originalHO-DiCBamount}\times 100$$

### 2.8. Statistical Analysis

For each assay duplicate or triplicate measurements were conducted.

## 3. Results and Discussion

### 3.1. Characterization of MCCBs

**Figure 1:**
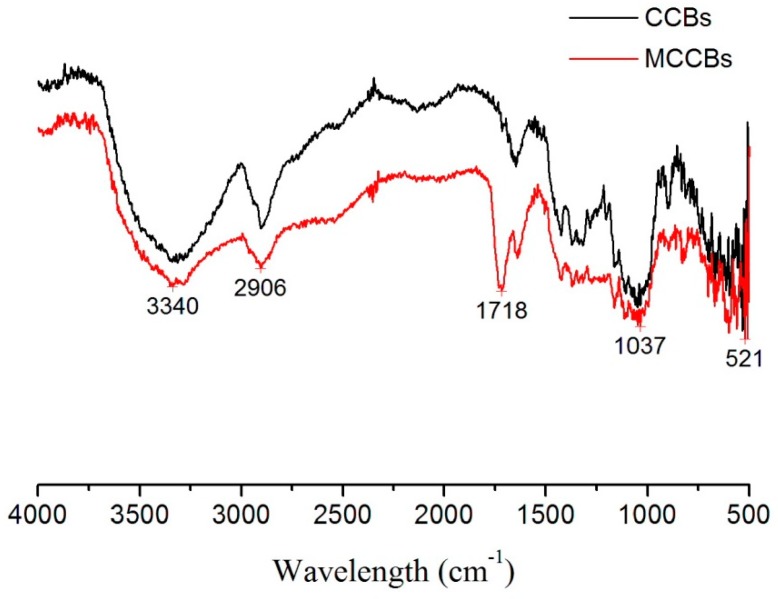
FT-IR of CCBs and MCCBs.

Figure 1 presents the FTIR spectra of CCBs and MCCBs. The peak at 3340 cm^−1^ is assigned to –OH group, and the strong absorption at 2906 cm^−1^ is due to C–H stretching from cellulose. When the FTIR spectra of MCCBs were compared to that of CCBs, there was a strong absorption peak at 1718 cm^−1^, which corresponds to the C=O bonds. The FTIR spectra showed that the maleic anhydride was grafted onto the cellulose surface successfully. In order to determine the amount of the carboxyl groups, a titration method was used and the concentration of carboxyl groups of MCCBs was 0.519 mmol/g.

**Figure 2:**
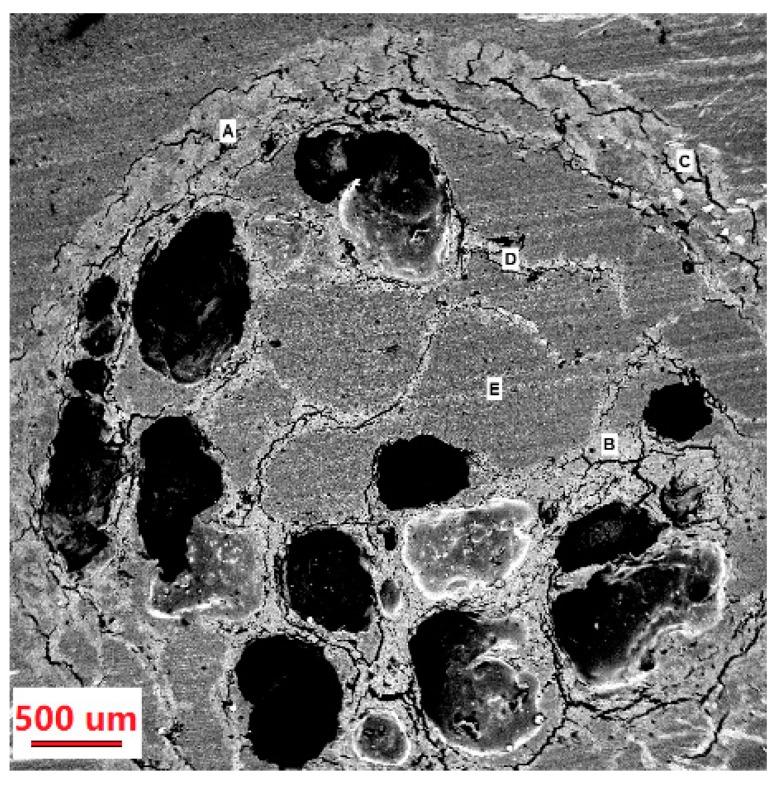
Scanning electron micrographs of cross-section MCCBs. A, B and C are cellulose; D and E are CF.

The cross-section of MCCBs was observed using SEM; the results are shown in Figure 2. Multiple small hollow pockets were found in Figure 2, indicating that the beads with porous structure were obtained using the method described previously. The small hollow pockets were formed probably due to the generation and release of the CO_2_ originating from nano-sized calcium carbonate as a pore forming agent.

### 3.2. Optimum Laccase Immobilization Conditions

#### 3.2.1. Effect of pH on Laccase Immobilization

To research the effect of pH on the laccase immobilization, 10 mg support was put into 8 g/L laccase solutions with pH ranged from 3.0 to 7.0 at 25 ± 1 °C and contact time was 2 h. Generally, the strength of the acting force between the support and laccase during an adsorption process was decided by the charge properties of the support surface and the laccase molecule [26]. However, laccase enzyme shows different charge characteristics under different pH conditions, and the change of the electrostatic association will influence the recovered activity and laccase immobilization yield [27].

**Figure 3:**
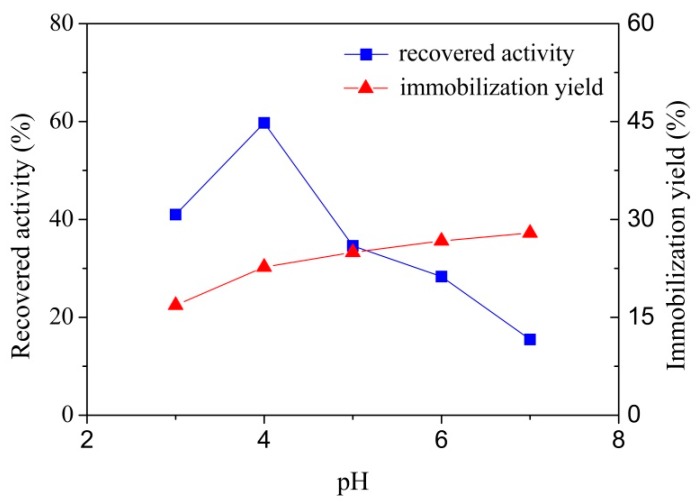
Effect of pH on immobilized laccase recovered activity and laccase immobilization yield by adsorption on MCCBs.

The immobilization yield was increased along with pH as shown in Figure 3. This is probably because when pH was higher than the laccase isoelectric point, laccase molecules and support were bridged by residual calcium ions. However, the recovered activity did not show a monotone increase trend, and the highest immobilized laccase recovered activity was achieved at pH 4.0. This result illustrated that pH significantly influenced the laccase activity, which agreed well with previous studies [28,29]. The laccase activity can be altered by varying pH, which can change the configuration of protein [30,31]. Despite having a lower immobilization yield at pH 4.0, the recovered activity of immobilization laccase showed a significant advantage, and thus pH 4.0 was chosen in the following research.

#### 3.2.2. Effect of Initial Enzyme Concentration on Laccase Immobilization

**Figure 4:**
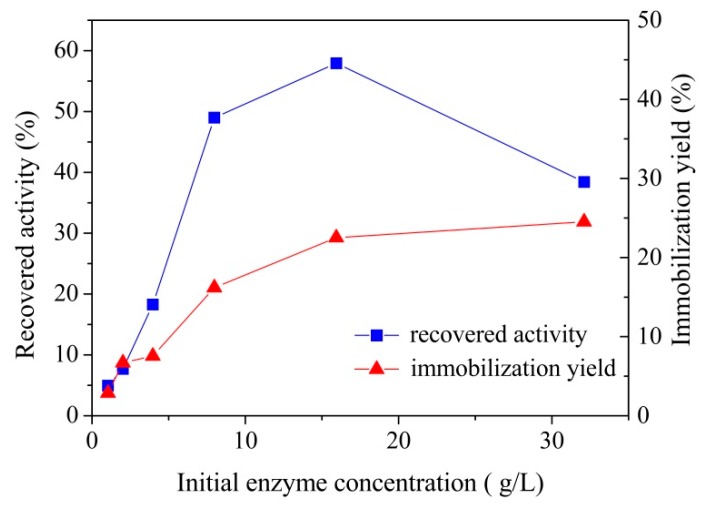
Effect of initial enzyme concentration on immobilized laccase recovered activity and laccase immobilization yield by absorption on MCCBs.

To find the optimum enzyme concentration, different concentration of enzyme solutions were used to immobilize laccase in a set of experiments. Under the optimum conditions, i.e., pH = 4 and *T* = 25 ± 1 °C, contact time 2 h, the effect of different enzyme concentrations on laccase immobilization yield was shown in Figure 4. The enzyme concentrations were varied from 0.5 to 32 g/L. As shown in Figure 4, the immobilization yield had a sharp increase with the increase of enzyme concentration and reached a high point at 16 g/L, followed by a slight increase as the concentration further increased to 32 g/L. This result indicated that the enzyme concentration has a significant effect on laccase immobilization yield, and high enzyme concentration had a promoting function on the reaction between laccase and the MCCBs support. This was because the high enzyme concentration could facilitate laccase to cover the entire surface of support, or even induce the multilayer adsorption [32]. Furthermore, this conclusion was in line with another study which considered that the incorporation of enzyme from solution onto support surface was clearly dependent on the enzyme concentration in solution [33].

On the other hand, the recovered activity of immobilized laccase was increased with increase of enzyme concentration and achieved the highest value at 16 g/L, which was consistent with the trend of immobilization yield, but the activity sharply decreased when the enzyme concentration further increased to 32 g/L. The highest recovered activity achieved at 16 g/L indicated that probably the laccase molecule covered the entire support surface or adsorption reached the equilibrium at this concentration, and with the further increase of the enzyme concentration in solution, no more support surface would be available to adsorb laccase and the adsorption occurred between the first layer laccase and the laccase in solution [34]. In other words, when concentration was above 16 g/L, the excess laccase was loaded on top of other laccase to create multi-layer adsorpion. Under this condition, although more laccase was loaded on the support, the active sites of the first layer were covered by the second layer, which resulted in the decrease of recovered activity. 

According to these results, the optimum initial enzyme concentration should be 16 g/L, since it was the best choice after overall consideration of immobilization yields and recovered activity. Above this point, although there was a slight increase of the immobilization yield, the recovered activity showed a clear decline.

#### 3.2.3. Effect of Contact Time on Laccase Immobilization

**Figure 5:**
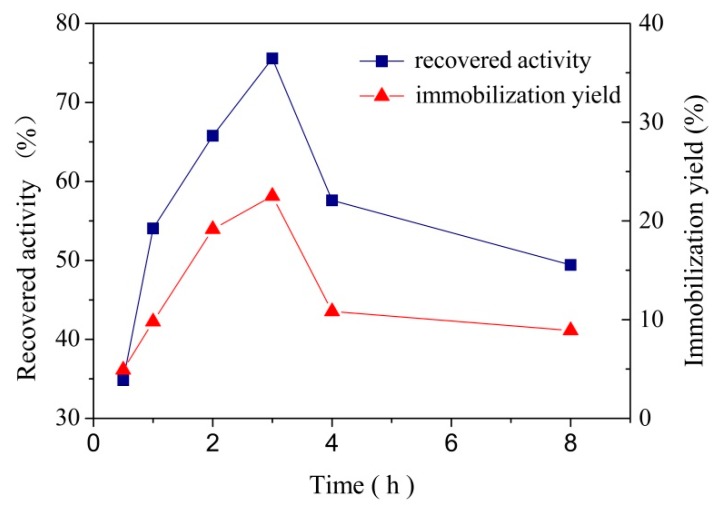
Effect of contact time on immobilized laccase recovered activity and laccase immobilization yield by absorption on MCCBs.

Contact time is another important factor in laccase immobilization. The effect of contact time was evaluated by putting 10 mg MCCBs into 16 g/L laccase solution under pH 4.0, *T* = 25 ± 1 °C conditions. The contact time was set from 0.5 to 8 h and the effect of contact time on recovered activity and laccase loaded were showed in Figure 5.

It was observed that the laccase immobilization yield increased with the increase of the contact time until 3 h and then showed a decline. This result illustrated that at 3 h the laccase reached the maximum adsorption. After 3 h, the laccase immobilization yield decreased, probably because of desorption, and the same result was also found in other studies [32,35]. The curve of recovered activity changed in line with that of laccase immobilization yield. In general, there was a positive correlation between the laccase immobilization yield and recovered activity. So the highest recovered activity achieved at 3 h also confirmed that at this point the maximum adsorption load was reached. Based on the data mentioned above, it could be concluded that the optimum contact time was 3 h and there was no advantage to prolong the contact time.

### 3.3. EDS Analysis of Immobilized Laccase MCCBs

**Figure 6:**
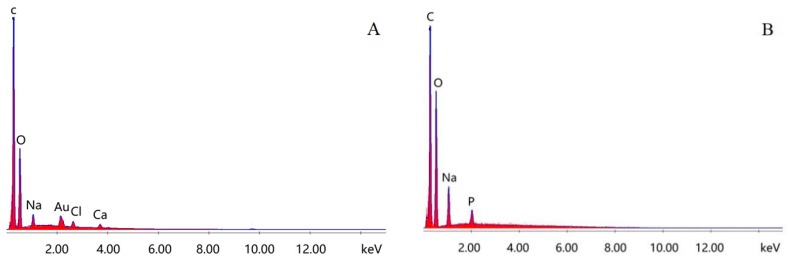
EDS spectra of the MCCBs (**A**) and immobilized laccase MCCBs (**B**).

The energy dispersive X-ray spectra (EDS) of the MCCBs and immobilized laccase MCCBs are presented in Figure 6. Compared to Figure 6A, P signal appeared in Figure 6B, indicating the presence of the laccase on the MCCBs. 

### 3.4. Thermal and Operational Stability of Immobilization Laccase

**Figure 7:**
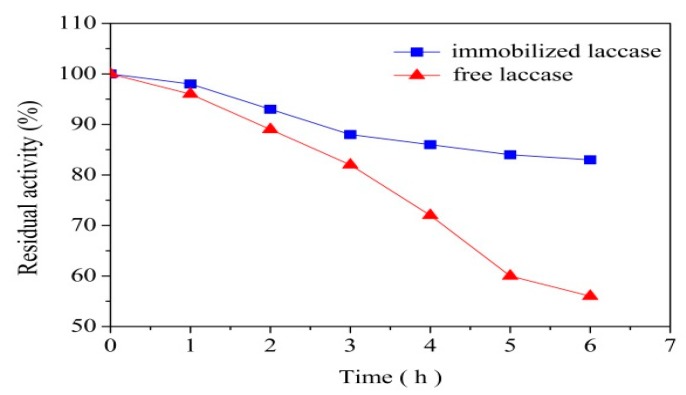
Thermal and operational stability of laccase immobilized on MCCBs by absorption at pH 4.0, 16 g/L initial lacasse concentration and 3 h contact time.

The thermal and operational stabilities are the two most important parameters related to the application of the immobilized laccase. The thermal stability (left) and operational stability (right) are shown in Figure 7. To evaluate the thermal stability, the residual activities of free and immobilized laccase were measured at 60 °C for 6 h. The immobilized laccase was obtained at the optimum experiment conditions: enzyme solution concentration 16 g/L, pH 4.0 and contact time 3 h. As can be seen from Figure 7 (left), the thermal stability of immobilized laccase is significantly higher comparing to that of free laccase at 60 °C. At the end of the experiment, the immobilized laccase maintained 83% of its initial activity and only 56% was retained by free laccase. A similar result was also reported elsewhere [36]. The increase of immobilized laccase activity was due to the limited freedom of the immobilized laccase, which could decrease the chance of drastic conformational changes and increase the stability of the laccase [37]. Another possibility is that immobilization makes heated laccase, which has exposed its hydrophobic core, unlikely to form intermolecular aggregates [38].

Compared to free laccase, reusability was an advantage of immobilized laccase, which has a great influence on cost saving. The immobilized laccase obtained at the optimum conditions was studied by cycles of ABTS oxidation, and 10 cycles were carried out to evaluate the operational stability. The results are shown in Figure 7 (right). After five cycles the immobilized laccase lost about 37% of initial activity. The loss of the activity was attributed to part of immobilized laccase with weak binding force desorbed from the MCCBs support during the washing process. After that, the residual activity showed a stable trend which retained about 60% of initial activity after six cycles. Although some results obtained in other studies were better than that in this work, which mainly because the support and immobilizing methods were different. Cellulose is a new kind of support in laccase immobilizing, and the performance identified in this work demonstrated that the laccase immobilized with MCCBs is of great potential for practical application.

### 3.5. Degradation of PCBs by Immobilized Laccase

**Figure 8:**
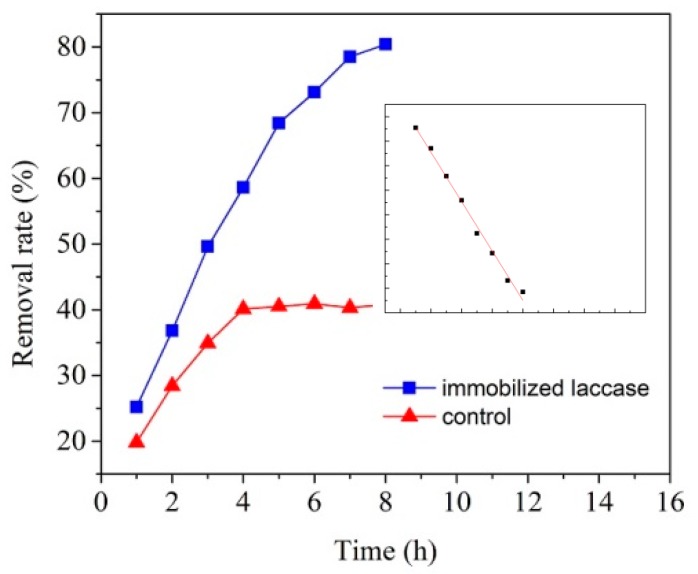
Removal rate of HO-DiCB by immobilized laccase and control (CCBs without laccase) at pH 4.0 room temperature; inlet: ln(1 − *r*) vs. *t* plot of curve for immobilized laccase.

The activity of MCCBs immobilized laccase in degrading PCBs was evaluated in this study and the removal rate is shown in Figure 8. As can be seen from Figure 8, the removal rate of HO-DiCB by immobilized laccase was about 85% whereas the result obtained in the control experiment was about 44%. The degradation in the control experiment was probably due to the adsorption of HO-DiCB on MCCBs instead of biodegradation. The HO-DiCB transferred from the solution to the surface of the control resulted in the decrease of the concentration of the HO-DiCB; similar results of removing PCBs by adsorption methods were also reported by others [39,40]. The removal rate of immobilized laccase was obviously higher than that of control beads without laccase immobilized, indicating that HO-DiCB was effectively degraded by immobilized laccase. Moreover, considering the removal ability of the control beads, the high removal rate of the immobilized laccase probably resulted from both the enzymatic degradation and adsorption processes or synergetic effect of both processes. Although there were some researches on degrading PCBs by laccase, few results of removal rate had been reported yet. The result gained in this research illustrated that immobilizing laccase on MCCBs is promising for better degradation of PCBs. Clearly, a plateau occurred in the control curve, which might correspond to the maximum amount of HO-DiCB that can be absorbed onto MCCBs surface or inside pores. We simulated this dynamic curve with a two-reagent reaction model (Equation (4)) by fixing initial content of PCBs (*r*_0_) and adsorption capacity of MCCBs (*s*_0_) to 1 and 0.408 (the height of plateau) respectively, and reaction constant to 1. The results indicated the excellent fitting qualitatively. The laccase catalyzed HO-DiCB concentration curve was fitted with the first-order reaction shown below:

*t* = 1/*k* × 1/(*r*_0_ − *s*_0_) × ((ln(*r*_0_ − *x*)/*r*_0_) − ln((*s*_0_ − *x*)/*s*_0_)) (4)

ln(1 − *r*) = *A* − *kt* (5)

where *r* is the ratio of removal, *t* is time, *k* is reaction rate constant, and *A* is an offset factor.

The curve fits this equation perfectly. While *A* is supposed to be 0 when *r* and *t* both go to 0, our fitting suggests that *A* being zero introduced significant bias. This non-zero offset could be due to measurement uncertainty or residual adsorption. Noticeably, this linear curve did not show any turning point, indicating that laccase adsorption suppressed PCBs adsorption onto MCCBs due to covering of binding sites and PCBs degradation.

## 4. Conclusions

A novel type of support beads (MCCBs), prepared by maleic anhydride-modified cellulose and nano CaCO_3_ powder as pore forming agent, was successfully produced and used to immobilize laccase in attempt to degrade HO-DiCB. The MCCBs showed excellent performance in terms of the immobilization yield and the recovered activity when used to immobilize commercial laccase. The immobilized laccase was reusable and maintained high residual activity, leading to the very efficient degradation of HO-DiCB. As green-based material, MCCBs can be considered as an appropriate support for immobilizing laccase, and the technology developed in this work has a potential advantage in removal PCBs in wastewater.
